# Elderly-onset hereditary pulmonary alveolar proteinosis and its cytokine profile

**DOI:** 10.1186/s12890-017-0382-x

**Published:** 2017-02-17

**Authors:** Masayuki Ito, Kazuyuki Nakagome, Hiromitsu Ohta, Keiichi Akasaka, Yoshitaka Uchida, Atsushi Hashimoto, Ayako Shiono, Toshinori Takada, Makoto Nagata, Jun Tohyama, Koichi Hagiwara, Minoru Kanazawa, Koh Nakata, Ryushi Tazawa

**Affiliations:** 1 Bioscience Medical Research Center, Niigata University Medical and Dental Hospital, Niigata, 951-8520 Japan; 2 Department of Respiratory Medicine, Saitama Medical University, Saitama, Japan; 3 National Hospital Organization Nishi-Niigata Chuo Hospital, Niigata, Japan; 4 Department of Respiratory Medicine, Jichi Medical University, Tochigi, Japan

**Keywords:** Hereditary pulmonary alveolar proteinosis, GM-CSF, GM-CSF receptor, Elderly onset, Cytokine profile

## Abstract

**Background:**

Pulmonary alveolar proteinosis (PAP) is a rare lung disease characterized by surfactant accumulation, and is caused by disruption of granulocyte/macrophage colony-stimulating factor (GM-CSF) signaling. Abnormalities in CSF2 receptor alpha (CSF2RA) were reported to cause pediatric hereditary PAP. We report here the first case of *CSF2RA*-mutated, elderly-onset hereditary (h) PAP.

**Case presentation:**

The patient developed dyspnea on exertion, and was diagnosed with PAP at the age of 77 years, based on findings from chest computed tomography scan and bronchoalveolar lavage. She tested negative for GM-CSF autoantibodies, with no underlying disease. Her serum GM-CSF level was elevated (91.3 pg/mL), indicating GM-CSF signaling impairment and genetic defects in the GM-CSF receptor. GM-CSF-stimulated phosphorylation in signal transducer and activator of transcription 5 (STAT5) was not observed, and GM-CSF-Rα expression was defective in her blood cells. Genetic screening revealed a homozygous, single-base C > T mutation at nt 508—a nonsense mutation that yields a stop codon (Q170X)—in exon 7 of *CSF2RA*. High-resolution analysis of single nucleotide polymorphism array confirmed a 22.8-Mb loss of heterozygosity region in Xp22.33p22.11, encompassing the *CSF2RA* gene. She was successfully treated with whole lung lavage (WLL), which reduced the serum levels of interleukin (IL)-2, IL-5, and IL-17, although IL-3 and M-CSF levels remained high.

**Conclusions:**

This is the first known report of elderly-onset hPAP associated with a *CSF2RA* mutation, which caused defective GM-CSF-Rα expression and impaired signaling. The analyses of serum cytokine levels during WLL suggested that GM-CSF signaling might be compensated by other signaling pathways, leading to elderly-onset PAP.

**Electronic supplementary material:**

The online version of this article (doi:10.1186/s12890-017-0382-x) contains supplementary material, which is available to authorized users.

## Background

The lungs reduce alveolar surface tension by producing pulmonary surfactants, which are essential for maintaining smooth breathing. The levels of pulmonary surfactants are balanced between their production by alveolar epithelial cells and clearance by alveolar macrophages. Granulocyte/macrophage colony-stimulating factor (GM-CSF), a 23-kDa monomeric glycoprotein, regulates various functions of the alveolar macrophages, including surfactant catabolism, and plays an important role in pulmonary surfactant homeostasis.

Pulmonary alveolar proteinosis (PAP) is a rare lung disease characterized by the accumulation of surfactant proteins, which causes progressive respiratory insufficiency [1–3]. Three clinically and etiologically distinct forms of PAP are acknowledged: autoimmune, secondary, and congenital. More than 90% of PAP cases are autoimmune type (aPAP). aPAP is specifically associated with high levels of a neutralizing autoantibody against GM-CSF that impairs GM-CSF-dependent surfactant clearance mediated by alveolar macrophages [4–8].

The GM-CSF signal is conveyed through the GM-CSF receptor on cell surfaces, via signal transducer and activator of transcription 5 (STAT5). The GM-CSF receptor is a dodecameric complex consisting of a ligand-specific alpha (GM-CSF-Rα, CD161) and a beta (GM-CSF-Rβ, CD131) subunit, which is shared with the interleukin (IL)-3 and IL-5 receptors [9, 10].

Recently, it has been reported that abnormalities in the GM-CSF-Rα-encoding alleles (*CSF2RA*) cause hereditary (h) PAP, presenting as insidious, progressive dyspnea in children; and that increased serum GM-CSF is useful to identify such individuals [11–15]. However, no elderly-onset case with defect in the GM-CSF receptor was reported until date. In the present report, we describe the oldest case of hPAP until date, whose symptoms occurred at the age of 77. The patient suffered severe hypoxemia, underwent a single whole lung lavage (WLL), and showed dramatic improvement.

## Methods

### Measurement of GM-CSF autoantibody levels

The concentration of GM-CSF autoantibodies in the bronchoalveolar lavage fluid (BALF) or serum were measured by sandwich enzyme-linked immunosorbent assay (ELISA), performed as described previously [16, 17], with slight modifications. In brief, 96-well microtiter plates (Maxisorp, Nalge Nunc International, Rochester, NY, U.S.A.) were coated with recombinant human (rh) GM-CSF [1 μg/mL in phosphate-buffered saline (PBS)] at 4 °C overnight, washed with PBS (containing 0.1% Tween 20), and blocked with Stabilcoat (Surmodics, Eden Prairie, MN, U.S.A.) at room temperature for 1 h. The BALF sample was diluted to 1/100 (for patients with PAP) in sample dilution buffer [PBS, 1% (w/v) goat serum, 0.1% (v/v) Tween 20], and 50 μL of the diluted sample was incubated in triplicate wells at room temperature for 40 min. The plates were washed and after addition of ammonium acetate (10 mM, pH 5.0), incubated at room temperature for 15 min to prevent non-specific binding. Next, horseradish peroxidase-conjugated goat anti-human IgG (diluted to 1/3000 with sample dilution buffer) was added, and the plates incubated at room temperature for 30 min. Bound IgG was detected using 3,3,5,5-tetramethylbenzidine substrate solution (50 μL; Bethyl Laboratories, Montgomery, TX, U.S.A.), followed by addition of 1 N H_2_SO_4_. The absorbance was read at 450 nm using a microplate reader (Bio-rad, Hercules, CA, U.S.A.).

### Immunoblotting

Peripheral blood mononuclear cells (PBMCs) were cultured on 24-well culture plates at 5 × 10^5^ cells/well. The cells were incubated with GM-CSF (0–1000 ng/mL) for 15 min. Lysed protein extracts were separated by sodium dodecyl sulphate polyacrylamide gel electrophoresis, transferred to a polyvinylidene fluoride membrane, and assessed by standard western blotting procedures, as previously described [5]. Primary antibodies used for detection included anti-human GM-CSF-Rα (Santa Cruz Biotechnology, Santa Cruz, CA, U.S.A.), GM-CSF-Rβc, anti-human STAT5 (Santa Cruz Biotechnology) and phospho (p)-STAT5 (Millipore, Billerica, MA, U.S.A.) antibodies. Peroxidase-labeled anti-rabbit IgG antibody (Sigma-Aldrich, St. Louis, MO, U.S.A.) was used as secondary antibody and the bands were visualized with ECL plus (GE Healthcare, Waukesha, WI, U.S.A.). To enhance the signal, immunoreaction enhancer solution (Can Get Signal, TOYOBO, Osaka, Japan) was used according to the manufacturer’s instructions. Actin was measured as loading control for each sample, using anti-actin antibody (Santa Cruz Biotechnology).

### Flow cytometry

Phosphorylated STAT5 detection by flow cytometry was described previously [18]. Heparinized fresh whole blood was incubated with 50 ng/mL rhGM-CSF and IL-2 for 20 min at 37 °C and fixed. Next, red blood cells were lysed in Fix/Lyse buffer (BD Bioscience, Franklin Lakes, NJ, U.S.A.) for 15 min at 37 °C. White blood cells were collected by centrifugation and fixed in ice-cold methanol at −20 °C for 1 h. After centrifugation, the cells were suspended in 3% fetal bovine serum/0.01% NaN_3_/PBS solution and incubated with Alexa Fluor 647-labeled anti-pSTAT5 (BD Bioscience). Cells with phosphorylated STAT5 were detected by flow cytometry (Cell Analyzer, Sony, Tokyo, Japan).

### Reverse transcription polymerase chain reaction (RT-PCR)

Total RNA was extracted from blood mononuclear cells, using RNA Easy Plus Mini Kit (QIAGEN, Hilden, Germany), and was reverse transcribed with random hexamer primers and the SuperScript III First-Stand Synthesis System for RT-PCR (Invitrogen, Carlsbad, CA, U.S.A.). The cDNAs were subjected to semiquantitative RT-PCR analysis with PrimerSTAR GXL DNA polymerase (Takara Bio, Otsu, Japan) with *CSF2RA*-specific primers (Additional file 1: Table S1).

### Nucleotide sequencing

The PCR was performed to generate products spanning exons and flanking sequences of the *CSF2RA* gene, which were purified using the QIAquick DNA extraction kit (QIAGEN) and subjected to nucleotide sequencing using the BigDye Terminator v3.1 cycle sequencing kit (Applied Biosystems, Foster City, CA, U.S.A.) and *CSF2RA*-specific primers (Supplementary Table). The resulting sequences were compared with published sequences for *CSF2RA* (GenBank/EMBL/DDBJ under accession number NM_006140).

### Array-comparative genomic hybridization (aCGH)

aCGH analysis was performed using the CytoScan HD array kit according to the manufacturer’s protocol (Affymetrix, Santa Clara, CA, U.S.A.). Genetic counseling was performed for the patient before and after genetic analyses.

### Cytokine assay

IL-2, IL-3, IL-4, IL-5, IL-17, and M-CSF were analyzed using ELISA kits (CUSABIO, Wuhan, Hubei Province, P.R. China) according to the manufacturer’s protocol.

### Case presentation

A previously healthy, 77-year-old, non-smoking woman, with normal chest radiograph at a medical check-up three years earlier, was referred to our hospital for dyspnea on exertion, which occurred one month earlier. She was a homemaker with no remarkable family history.

**Fig. 1:**
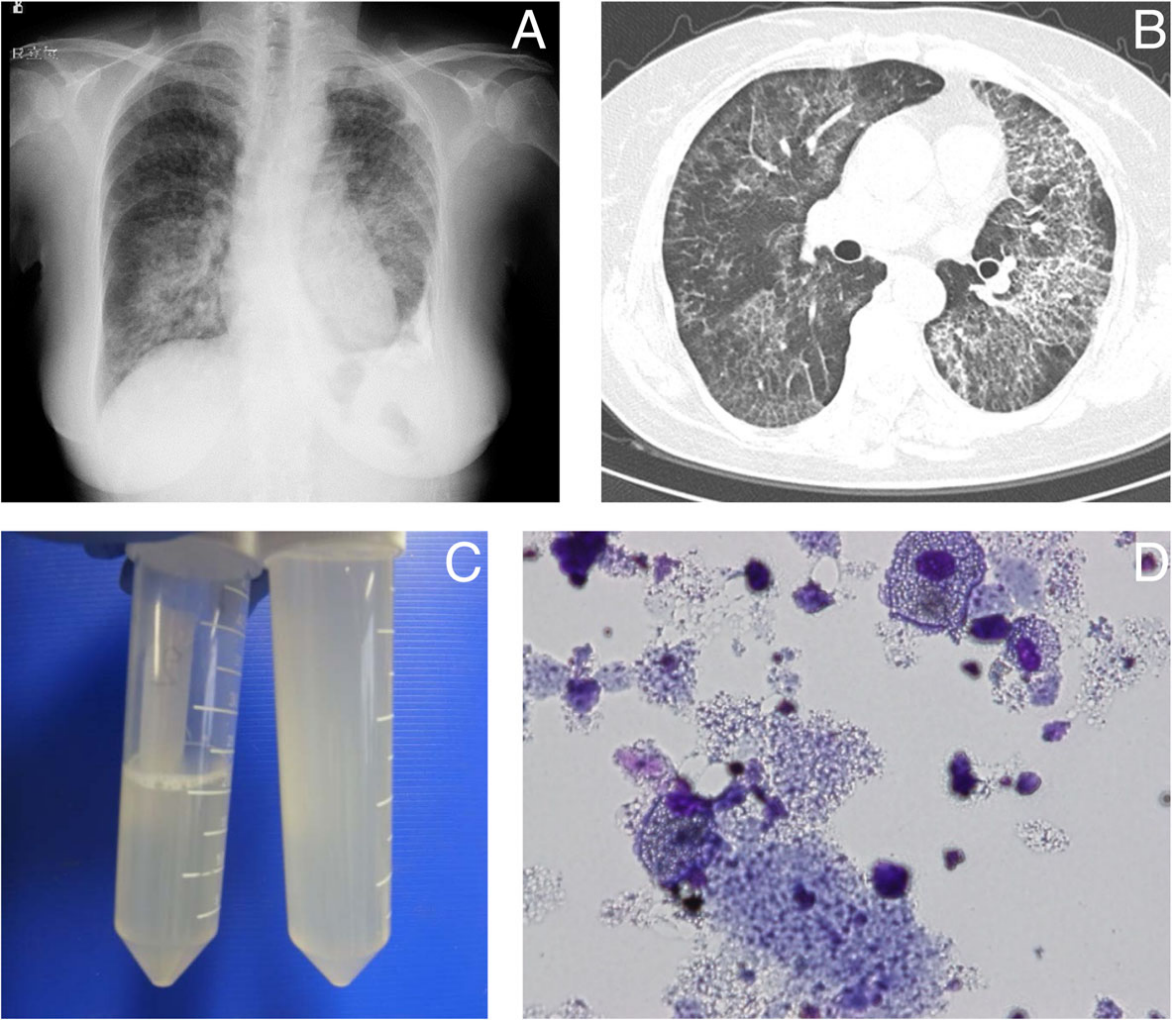
**a** Chest radiogram of the patient. **b** High-resolution computed tomogram of the patient. **c** BALF presented a milky appearance with lymphocytosis (26%). **d** Foamy macrophages and amorphous materials in BALF of the patient

A physical examination revealed no abnormality. A chest X-ray and computed tomography (CT) scan indicated ground glass opacity in both lower lungs, of which crazy-paving appearance was confirmed upon high-resolution CT scan (Fig. 1a, b). Laboratory studies revealed elevated levels of serum KL-6, a mucin-like protein (4314 U/mL), surfactant protein D (SP-D; 400 ng/mL), and carcinoembryonic antigen (CEA; 13.2 ng/mL); and negative test results for beta-D glucan and Aspergillus antigen. Analysis of arterial blood gas while breathing room air revealed a low partial pressure of oxygen (PaO_2_) of 47 mmHg just before WLL. BALF presented a milky appearance with lymphocytosis (macrophages, 64% of total cells; lymphocytes, 26%; neutrophils 9%; eosinophils, 1%), foamy macrophages, and amorphous materials (Fig. 1c, d).

The patient was diagnosed with PAP, based on typical findings from chest CT scan and bronchoalveolar lavage. She had no underlying disease. GM-CSF autoantibodies were not detected in either the serum or the BALF. On the other hand, a high level of serum GM-CSF was detected (91.3 pg/mL), indicating disrupted GM-CSF signaling and genetic defects in the GM-CSF receptor. We decided to screen for GM-CSF signaling abnormalities because GM-CSF concentration was extremely high in her BALF.

**Fig. 2:**
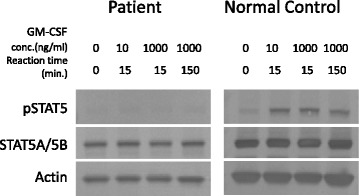
Granulocyte/macrophage colony-stimulating factor (GM-CSF)-stimulated phosphorylation of signal transducer and activator of transcription 5 (STAT5) in peripheral blood mononuclear cells (PBMCs) of the patient. PBMCs from either the patient or a normal control subject were incubated with different concentrations of GM-CSF (0–1000 ng/mL). Phosphorylated (p) STAT5 was detected by western blotting. Total STAT5 (STAT5) and actin levels were examined as positive controls

**Fig. 3:**
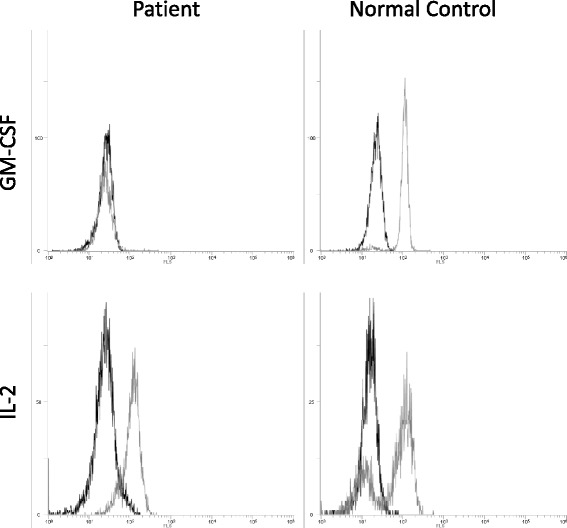
Flow cytometry analysis for the expression of phosphorylated STAT5 upon stimulation of peripheral blood mononuclear cells of the patient (left) and a control (right) with interleukin (IL)-2 and granulocyte/macrophage colony-stimulating factor (GM-CSF)

To investigate signaling activated by GM-CSF in the PBMCs, we first probed the presence of phosphorylated STAT5 upon stimulating the PBMCs with increasing concentrations of GM-CSF (0–1000 ng/mL). Phosphorylated STAT5 was observed in the control but not in the patient PBMCs, indicating defective GM-CSF signaling in the latter (Fig. 2). To confirm this result, we performed flow cytometry. The results also showed that expression of phosphorylated STAT5 was not observed on stimulation of peripheral blood mononuclear cells of the patient with GM-CSF, while expression of phosphorylated STAT5 was observed on stimulation of those of the control with GM-CSF as well as on stimulation of those of both with IL-2 (Fig. 3).

**Fig. 4:**
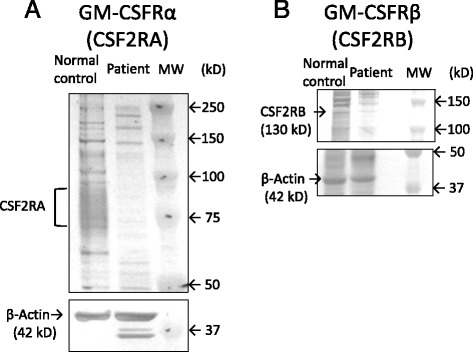
**a** Detection of granulocyte/macrophage colony-stimulating factor receptor alpha (GM-CSFRα; upper panel), and actin (lower panel) in peripheral blood monocytic cells of the patient (PBMCs) (left), and a control (middle), by western blotting with specific antibodies. GM-CSF-Rα was not detected in the patient (left lane) but was observed for the control (middle lane). **b** Detection of GM-CSFRβ (upper panel) and actin (lower panel) in PBMCs of the patient (left), and a control (right), by western blotting with specific antibodies. Bands for GM-CSF-Rβ and actin were detected for both the patient and the control

**Fig. 5:**
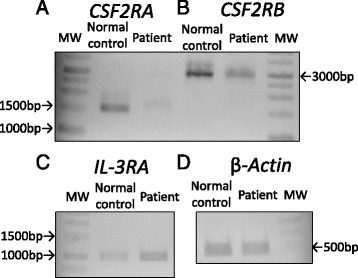
Reverse transcription polymerase chain reaction for mRNA obtained from peripheral blood mononuclear cells of the patient and a normal control, using primers specific for *CSF2A*, *CSF2B*, *IL3RA*, and *ACTB*

Next, we investigated the expression of the α and β chains of the GM-CSF receptor on the PBMCs, by western blotting. The expression of GM-CSF-Rβ and IL-3Rα on patient monocytes was comparable with that on the controls; however, expression of GM-CSF-Rα was not detected in the former (Fig. 4). The result was further confirmed by RT-PCR (Fig. 5). Together, these results indicated defects in the molecules that transmit GM-CSF signals to STAT5.

**Fig. 6:**
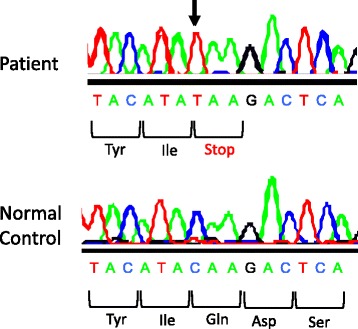
Nucleotide sequence of the *CSF2RA* gene, encoding nt 502–516 (numbered relative to the initiation codon; GenBank/EMBK/DDBJ accession no. NM_006140), from the patient and a normal control

cDNAs of *CSF2RB,* reverse-transcribed from the mRNA of the patient PBMCs, harbored no mutations or deletions (data not shown). PCR amplicons for each exon of *CSF2RA* from the patient PBMCs were screened for their nucleotide sequences, revealing a homozygous single-base mutation C > T at nt 508 in exon 7 (Fig. 6). This mutation was a nonsense mutation that gave rise to a stop codon (Q170X).

Further evaluation by aCGH demonstrated a 22.8-Mb region of loss of heterozygosity (LOH) in Xp22.33p22.11, encompassing the *CSF2RA* gene.

**Fig. 7:**
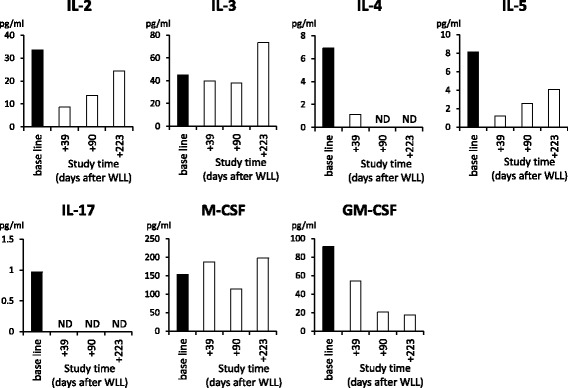
Analysis of cytokine profile of the patient before and after whole lung lavage, by enzyme-linked immunosorbent assay for interleukin (IL)-2, IL-3, IL-4, IL-5, IL-17, M-CSF, and GM-CSF

To investigate the functional background underlying the late onset of the symptoms in the present case, we compared the changes in serum levels of IL-2, IL-3, IL-4, IL-5, IL-17, and M-CSF before and after WLL that improved PaO_2_ by 26 mmHg (Fig. 7). The IL-2, IL-4, and IL-5 levels were remarkably reduced post WLL, after which they gradually increased. IL-17 levels remained low for about 7 months after WLL. On the other hand, IL-3 and M-CSF levels were marginally altered post WLL. In addition, the serum level of GM-CSF demonstrated a tendency to decrease after WLL.

## Discussion

The present study reports the first case of elderly-onset hPAP with *CSF2RA* mutation. The patient had a homozygous mutation that changed a codon for glutamine 170 to a stop codon, disrupting normal expression of GM-CSF-Rα and proper signaling of GM-CSF. We previously reported a case of adult-onset hPAP that was due to homozygous nonsense mutation in the *CSF2RB* gene [15]. The present case, where the patient developed hPAP in her seventies, is the oldest until date for this disease (Table 1). Interestingly, her mutation was in exon 7 of the *CSF2RA* gene, in which the first child case for hPAP also had a mutation [11].

**Table 1:** Clinical characteristics of reported cases of hereditary pulmonary alveolar proteinosis

	Present case	Tanaka et al.	Junne et al.
Characteristics	Adult onset	Adult onset	Juvenile (n = 20)
Sex	Female	Female	85% (Female)
Age at symptom onset (yr)	77	35	0.2–19
Prognosis	Good	Poor	Good
GM-CSF (pg/mL)	86.61	124.8	Elevated
GM-CSF antibody (μg/mL)	ND	ND	ND
Mutated gene	*CSF2RA*	*CSF2RB*	*CSF2RA*
WLL therapy	responded	Worsened	Improved 64%
			Unchanged 14%
			Worsened 0%
			Deceased 7%

*GM-CSF* granulocyte/macrophage colony-stimulating, *ND* not detected, *CSF2RA*, *CSF2* receptor alpha, *CSF2RB*, *CSF2* receptor beta, *WLL* whole lung lavage

To investigate the background of the late-onset hPAP in this case, we studied her serum profile for various cytokines. The patient was treated with WLL, which greatly improved oxygenation indices and reduced the serum levels of various cytokines including IL-2, IL-5, and IL-17, indicating relieved inflammatory activation of Th1 and Th17 lymphocytes. In contrast, the serum levels of IL-3 and M-CSF remained high after WLL, indicating that they might be needed even after the treatment procedure. The high activities of IL-3 and M-CSF in the patient might be associated with the clinical course of her late-onset hPAP.

It is notable that GM-CSF, IL-3, IL-4, and IL-5 are coded by neighbor genes located at 5q31.1, and affect the functions of monocytes and macrophages. The serum levels of IL-4, IL-5, and GM-CSF decreased after WLL, while IL-3 levels remained high. Receptors of IL-3 and IL-5 share beta chains with that of GM-CSF. Disruption of the alpha chain of the GM-CSF receptor might affect stoichiometry of the common beta chains, presumably causing substantial alterations in IL-3 and IL-5 signaling. Interestingly, WLL differentially affected the levels of these cytokines in the serum of the patient in this study.

Similar to previous pediatric hPAP cases, the present case also demonstrated a high level of serum GM-CSF and a good response to WLL (Table 1). The increased serum GM-CSF levels might be useful for identifying patients with childhood as well as adult hPAP [19, 20]. The present case showed remarkable response to WLL with regard to oxygenation indices and serum levels of various cytokines that might be associated with compensation of GM-CSF signaling.

## Conclusions

This is the first known report of elderly-onset hPAP associated with a genetic defect in *CSF2RA*, which caused defective GM-CSF-Rα expression and impaired signaling. These results suggest that GM-CSF signaling is compensated by other signaling pathways, leading to elderly-onset or non-symptomatic PAP.

## Additional file

Additional file 1: Table S1. A list of primers for PCR and sequencing. (DOCX 17 kb)

## Abbreviations

CSF2RA: Colony stimulating factor 2 receptor alpha chain
ELISA: Enzyme-linked immunosorbent assay
GM-CSF: Granulocyte/macrophage–colony stimulating factor
hPAP: Hereditary PAP PAP, pulmonary alveolar proteinosis
IL-2: Interleukin2
