# Supplementary material for: Elderly-onset hereditary pulmonary alveolar proteinosis and its cytokine profile
Source: BMC Pulm Med. 2017 Feb 17;17:40. doi: 10.1186/s12890-017-0382-x (PMC5316164; doi:10.1186/s12890-017-0382-x)
Supplement: Additional file 1: Table S1. — A list of primers for PCR and sequencing. (DOCX 17 kb) [file 12890_2017_382_MOESM1_ESM.docx]

Additional file: Table S1. A list of primers for PCR and sequencing

| gene |  | Primer Sequence |
| --- | --- | --- |
| *CSF2RA*-Exon1 | S* | 5'-CAATGAACTCACGGAGCAATTAC-3' |
|  | AS** | 5'-CTCCAACAACACCTCCCTCTTTTTA-3' |
| *CSF2RA*-Exon2 | S | 5'-CCCAGCCCTGGTATGTAATTTT-3' |
|  | AS | 5'-TCCTGGTTCATCATTATGGAGCA-3' |
| *CSF2RA*-Exon3 | S | 5'-CCTTGAAGTGTGTTAGAACCTGCC-3' |
|  | AS | 5'-CCAGATGCAGGACATCTAAACAGG-3' |
| *CSF2RA*-Exon4 | S | 5'-GAGGAAATTCTGAACCCAG-3' |
|  | AS | 5'-GGTGTGTAAAGGACAGGGG-3' |
| *CSF2RA*-Exon5 | S | 5'-GTAGGAGACAGAAGGTTGTTTCCTA-3' |
|  | AS | 5'-CTGGGAAGAGGTAGAAACAAAATAA-3' |
| *CSF2RA*-Exon6 | S | 5'-AGTACATCCCGTTGAACTTCGG-3' |
|  | AS | 5'-TCCCATCCCTGCATTCCTCATA-3' |
| *CSF2RA*-Exon7 | S | 5'-GTTTTCCTGATTGCTCTCTGAGC-3' |
|  | AS | 5'-CACCGCACGTGGCCTCAGTTAC-3' |
| *CSF2RA*-Exon8 | S | 5'-TCGGGTTCAGGGGTGTGTCCTGCGCCCTCG-3' |
|  | AS | 5'-GCCACAGACCCTTCACCTCTT-3' |
| *CSF2RA*-Exon9 | S | 5'-GACTCCTTCCCATTCGGTG-3' |
|  | AS | 5'-TGTCAGTAGGGTCTGTGGTTTCAC-3' |
| *CSF2RA*-Exon10 | S | 5'-AGACCAAGTGCATTCAGAGTGGTAG-3' |
|  | AS | 5'-CTTCTCTGGTTATTAAGGGAGCCAA-3' |
| *CSF2RA*-Exon11 | S | 5'-TGTCCGTCAACGATTCACCGCAGAC-3' |
|  | AS | 5'-AGACTGGACAAAGCGCTTTCCTCCC-3' |
| *CSF2RA*-Exon12 | S | 5'-TTGCATAGTTGAGCGCAGACA-3' |
|  | AS | 5'-CCATCTGGTGGAGAAGCAAGA-3' |
| *CSF2RA*-Exon13 | S | 5'-TGAAGATCTGACAGCCTGAACC-3' |
|  | AS | 5'-TCCAGCAATCCTCCAAAGTGT-3' |

Note: S* and AS** represent sense and antisense primers, respectively
